# Prevalence and associated factors of depression and anxiety among Chinese diabetic retinopathy patients: A cross-sectional study

**DOI:** 10.1371/journal.pone.0267848

**Published:** 2022-04-28

**Authors:** Ling Xu, Siqi Chen, Kai Xu, Yixin Wang, Hongda Zhang, Lie Wang, Wei He

**Affiliations:** 1 He Eye Specialist Hospital, Shenyang, China; 2 Department of Social Medicine, School of Public Health, China Medical University, Shenyang, Liaoning, People’s Republic of China; Chiba Daigaku, JAPAN

## Abstract

The purposes of this study were to evaluate the mental health among patients with diabetic retinopathy (DR) and to explore its’ influencing factors. A cross-sectional survey was conducted in Liaoning Province, China. A total of 200 patients volunteered to participate in the survey. Psycho-social variables included stress, self-efficacy, resilience, and social support. logistic regression analysis was used to explore the effect of psycho-social factors on depression and anxiety in DR patients. The prevalence of depression and anxiety was 25% and 13.5% among DR patients. Regression analysis showed that social support had a significant protective effect on depression (95%CI 0.931–0.997) and anxiety (95%CI 0.900–0.995). Stress had a significant acceleration of depression (95%CI 1.055–1.253) and anxiety (95%CI 1.120–1.457). Family history of diabetes (95%CI 1.856–18.057) and other chronic diseases (95%CI 1.485–12.508) were risk factors for anxiety. The high prevalence of depression (25.0%) and anxiety (13.5%) among DR patients should receive more attention in Chinese medical settings. Stress, social support, family history of diabetes and other chronic diseases seemed to be crucial in relation to depressive symptoms. Efficient interventions such as improving social support and decreasing stress with patients should be considered by health administrators aiming at alleviating depressive and anxiety among Chinese DR patients.

## Introduction

Diabetic retinopathy (DR) is one of the common complications of diabetes [1], which have a great harm. It has been estimated that one third of people with Diabetes Mellitus (DM) in the world have symptoms of DR, and about one in ten of them develop vision-threatening DR [2]. In China, the prevalence of DR is 18.2% in the high-risk diabetic population and 2.5% in the prediabetic population [3]. Because of the vitreous hemorrhage and tractional retinal detachment, DR patients tent to have vision loss. Furthermore, studies revealed that DR could disrupt circadian function [4] and cause neurocognitive defects [5] with the aggravation of symptoms.

Besides, a great number of studies reported that DR could contribute to mental disorders. Compared to diabetic patients without retinopathy, DR patients may be more vulnerable to suffer from the depression and anxiety [6]. According to a recent study, the prevalence of depression and anxiety in DR patients were 34.3% and 41.1% [2]. Depression and anxiety not only lead to a higher disease burden, but also accelerated disease progression for patients [7]. Furthermore, depression can lead to poorer diabetes control and lower treatment adherence [8].

Owing to the negative impacts of depression and anxiety among DR patients, increasingly studies have explored the variables influencing depression and anxiety among DR patients. In addition to the impacts of demographic and clinical variables on depression and anxiety [9–11], positive psychological variables started to receive increasing attention in oncology field over the last 20 year. According to the literature review, we found that depression and anxiety among patients with diabetes have been associated with factors such as poor social support [12], poor resilience [13], low levels of self-efficacy [14] and high stress [15]. And the correlation in the DR patients is being explored.

Social support has been defined as “a social network’s provision of psychological and material resources intended to benefit an individual’s ability to cope with stress” by Cohen [16]. Literature shows that social support plays a pivotal role in patients with diabetes [17] and provides support for the necessary physical and dietary changes [18]. Besides, previous study has reported that poorer social support was associated with greater levels of depression in DR patients [19].

Resilience is defined as manageable and measurable capability of a dynamic systemic to adjust successfully to adverse life experience, such as disturbances, frustrations, and failures that threaten the viability, the function, or the development of the system [20]. It has been found among participants with higher resilience had a lower probability of suffering from neuropathy as compared to participants with lower resilience [21]. Individuals with low resilience are associated with high levels of distress, poor quality of life, uncontrolled blood sugar, and nonadaptive coping mechanism [22].

Self-efficacy refers to a relatively stable belief of personal competence to deal effectively with a variety of stressful situations [23]. If an individual has a strong perceived self-efficacy, they will set high goals, be firmly committed to achieving these goals and be able to persist with considerable effort in the face of difficulties in achieving their goals [24–26]. Besides, study indicated that self-efficacy had a direct effect on diabetes self-care practice [27].

Stress is defined as the “wear and tear” the body experiences as it adjusts to pressure or a threating situation [28]. Study has shown that stress can promote the development of negative emotion and mental disorders, which not only affects the treatment compliance of patients, but also affects the prognosis and quality of life among patients with diabetes mellitus [29].

To date, studies in the diabetes have mainly explored the impact of the positive psychological variables on depression and anxiety among DR patients, few studies investigated the association of stress with negative emotion among Chinese DR patients. Therefore, the present study aimed to investigate the prevalence of depression and anxiety and explore the relationships between stress, social support, resilience, self-efficacy and depression and anxiety in patients with DR. More importantly, we aimed to confirm the integrative effects of stress, social, resilience and self-efficacy on depression and anxiety after adjusting for the demographic and clinical variables.

## Method

### Design and participants

In the current study, a cross-sectional design was carried out from March 2019 to May 2020. A total of 242 patients with DR were recruited from the He Eye Specialist Hospital of Liaoning Province through a convenience sampling. Inclusion criteria in this study were that participants (1) were less than 80 years old, (2) were diagnosed with DR, (3) were able to communicate in Chinese language well enough to answer the questionnaires, (4) had clear consciousness and cognition. Exclusion criteria were the following those patients (1) had a history of psychiatric problems (2) had other serious diseases (such as cancers). (3) had intellectual impairments. This study was approved by the Ethics Committee on Human Experiments of He Eye Specialist Hospital and was in conformity with the ethical standards (IRB AF/07.08/02.1). We got written information from questionnaires. When questionnaires are distributed, patients were asked to read the informed consent form and those who agree to participate in the survey will sign at the bottom. All self-report questionnaires were distributed to DR patients after obtaining the informed consent, and their individual privacy was protected. When investigating minors, we obtained consent from parents or guardians. We will only investigate a minor if their parents or guardians read and sign the informed consent form. Authors could not identify in dividual participants during and after data collection. Finally, 200 participants completed investigation, and effective response rate was 82.6%.

### Demographic and clinical data

We collected these data through a self-reported questionnaire. The following demographic and clinical characteristics were included: gender (man / woman), age (≤ 45 / 45–60 / ˃ 60, year), residence (rural/urban), monthly income (≤4000 / ˃4000, yuan). The following clinical characteristics were assessed: smoking (yes / no), drinking (yes / no), exercise (never / ≤ 3 times a week / > 3 times a week), family history of diabetes (yes / no), other chronic diseases: hypertension, heart disease, cerebrovascular disease, hyperthyroidism, tumor, neurasthenia and others (yes / no), blood glucose control (good HbAlC ≤ 6.2% / general 6.2% ≤ HbAlC ≤ 8.0% / bad HbAlC >8.0%), diabetes types (type 1 diabetes / type 2 diabetes / other types).

### Measurement of depression

In this study, we used the Patient Health Questionnaire (PHQ-9) to measure depression, which is composed of 9 items. The scale uses 4-level Likert score, ranging from 0 (never) to 3 (everyday). The total score ranges from 0 to 27. When the score is ≥ 10 points, it is considered as depression. The Chinese version of the PHQ-9 has been confirmed to have good reliability and validity [30]. The Cronbach’s α of the scale was 0.834 in the current study.

### Measurement of anxiety

In this study, we used the Generalized Anxiety Disorder (GAD-7) scale to measure anxiety. GAD-7 has 7 symptom items to assess the severity and functional impact of 7 anxiety symptoms in the last 2 weeks. The scale uses 4-level Likert score, ranging from 0 (never) to 3 (everyday). The total score ranges from 0 to 21. Anxiety exists when the score is ≥ 10. The Chinese version of GAD-7 has been widely utilized to assess the severity of anxiety symptoms in the Chinese populations [31]. The Cronbach’s α of the scale was 0.938 in this study.

### Measurement of stress

A 10-item Perceived Stress Scale (PSS) developed by Cohen et al [32] is used to measure the stress feelings in the past month. The scale uses 5-level Likert score, ranging from 0 (never) to 4 (always). The total score of the scale ranges from 0 to 40, with a higher score indicating a greater sense of pressure. The Chinese version of the PSS has demonstrated reliability and validity in China cancer patients [33]. The Cronbach’s α of the PSS was 0.684 in the present study.

### Measurement of self-efficacy

The General Self-Efficacy Scale (GSES) selected for this study was compiled by Schwarzer et al [34]. The scale uses 4-level Likert score, ranging from 1 (not at all true) to 4 (exactly true). It consists of 10 items and the total score ranges from 10 to 40. The higher the total score, the higher the level of self-efficacy of the patients. The Chinese version of GSES has been validated in Chinese clinical patients [33]. In this study, the Cronbach’s α was 0.941.

### Measurement of resilience

In this study, we used Resilience Scale (CD-RISC) which developed by Connor and Davidson [35]. The scale uses 5-level Likert score, ranging from 0 (never) to 4 (always). It consists of 25 items and the total score is 0–100. The higher the total score, the higher the level of resilience of the patients. CD-RISC has been translated into many languages and validated in previous studies, including Chinese populations [36]. In this study, the Cronbach’s α was 0.934.

### Measurement of social support

The level of social support in DR patients was measured by the Perceived Social Support Scale (PSSS) which developed by Zimet [37]. The scale uses 7-level Likert score, ranging from 1 (disagree absolutely) to 7 (agree absolutely). The scale consists of 12 items and the total score is 12–84. The higher the total score, the higher the level of social support of the patients. The Chinese version of the PSSS was shown to have adequate reliability and validity for other cancer patients [38]. The Cronbach’s α of the scale was 0.948 in this study.

### Statistical analysis

Statistical analysis was performed in SPSS 21.0. In the present study, the mean and standard deviation (SD) were used to describe the continuous variables, and categorical variables were described as a number and percentage (%). We used the Pearson chi-square test to analyze categorical variables, and *t*-test was used to compare continuous variables. Binary logistic regression analysis was performed for all variables to assess odds ratio (OR) and 95% confidence intervals (CI) to explore risk and protective factors affecting negative emotion in patients with DR. In this study, negative emotions (including depression and anxiety) were taken as the dependent variable and divided into two categories: patients with negative emotions and patients without negative emotions. Stress, social support, resilience and self-efficacy was regarded as independent variables. Age and gender were considered as fixed factor, and a regression model was developed to control potential founders according to univariate analysis (*P* < 0.25). Two-sided test was used in regression model with *P* < 0.05 was considered statistically significant.

## Results

### Demographic and clinical variables and depression and anxiety

The participants (n = 200) were in the age range of 25–79, and mean (SD) age of patients was 56.79(11.57) years old. 51.0% of these patients were females.

Demographic and clinical characteristics is shown in Table 1. Based on the cut-off values recommended of depression, 25% of these were patients with depression, 75% were patients without depression. Patients with depression and patients without depression significantly differed in terms of other chronic diseases (*P* < 0.05). In addition, gender, family history of diabetes and blood glucose control also had statistical differences between two groups (*P* < 0.25). there were no significant differences in terms of age, residence, monthly income, smoke, drink, exercise and diabetes type.

**Table 1:** Demographic and clinical variables and depression and anxiety.

Variable	Without depression 150 (%)	Depression 50 (%)	*χ2*	*P*	Without anxiety 173 (%)	anxiety 27 (%)	*χ2*	*P*
Gender			3.228	0.072			3.066	0.080
Man	79 (52.7)	19 (38.0)			89 (51.4)	9 (33.3)		
Woman	71 (47.3)	31 (62.0)			84 (48.6)	18 (66.7)		
Age			1.770	0.413			5.614	0.057
< 45	25 (16.7)	12 (24.0)			30 (17.3)	7 (18.5)		
45–60	58 (38.7)	20 (40.0)			64 (37.0)	14 (39.0)		
> 60	67 (44.7)	18 (36.0)			79 (45.7)	6 (42.5)		
Residence			0.000	1.000			1.999	0.157
Rural	54 (36.0)	18 (36.0)			59 (34.1)	13 (48.1)		
Urban	96 (64.0)	32 (64.0)			114 (65.9)	14 (51.9)		
Monthly income			0.733	0.392			2.240	0.134
≤ 4000	95 (63.3)	35 (70.0)			109 (63.0)	21 (77.8)		
> 4000	55 (36.7)	15 (30.0)			64 (37.0)	6 (22.2)		
Smoke			0.029	0.866			0.727	0.521
No	95 (63.3)	31 (62.0)			107 (61.8)	19 (70.4)		
Yes	55 (36.7)	19 (38.0)			66 (38.2)	8 (29.6)		
Drink			0.007	0.932			0.135	0.831
No	95 (63.3)	32 (64.0)			109 (63.0)	18 (66.7)		
Yes	55 (36.7)	18 (36.0)			64 (37.0)	9 (33.3)		
Exercise			0.470	0.790			1.391	0.499
Never	55 (36.7)	21 (42.0)			63 (36.4)	13 (48.1)		
≤ 3 times a week	25 (16.7)	8 (16.0)			29 (16.8)	4 (14.8)		
> 3 times a week	70 (46.7)	21 (42.0)			81 (46.8)	10 (37.0)		
Diabetes history			1.333	0.248			5.075	0.024
No	89 (59.3)	25 (50.0)			104 (60.1)	10 (37.0)		
Yes	61 (40.7)	25 (50.0)			69 (39.9)	17 (63.5)		
Other chronic diseases			6.222	0.013			9.707	0.002
No	112 (74.7)	28 (56.0)			128 (74.0)	12 (44.4)		
Yes	38 (25.3)	22 (44.0)			45 (26.0)	15 (55.6)		
Blood glucose control			2.793	0.247			2.330	0.312
Good	25 (16.7)	5 (10.0)			28 (16.2)	2 (7.4)		
General	74 (49.3)	22 (44.0)			84 (48.6)	12 (44.4)		
Bad	51 (34.0)	23 (46.0)			61 (35.3)	13 (48.1)		
Diabetes type			1.359	0.507				
type 1 diabetes	4 (2.7)	3 (6.0)			5 (2.9)	2 (7.4)		
type 2 diabetes	121 (80.7)	40 (80.0)			142 (82.1)	19 (70.4)		
Other types	25 (16.7)	7 (14.0)			26 (15.0)	6 (22.2)		

Based on the cut-off values recommended of anxiety, 13.5% of these were patients with anxiety, 86.5% were patients without anxiety. Results indicated that patients with anxiety and patients without anxiety significantly differed in terms of family history of diabetes and other chronic diseases (*P* < 0.05). In addition, gender, age, residence and monthly income also had statistical differences in the patients with depression and patients without depression (*P* < 0.25)., there were no significant differences in terms of smoke, drink, exercise and blood glucose control.

### Relationships between psycho-social variables and depression and anxiety

The psycho-social variables are shown in Table 2. It shown that there was a statistical difference in the average scores for stress, self-efficacy, resilience and social support between patients with depression and patients without depression. Patients with depression had significantly higher scores than patients without depression for stress (*P* < 0.001) and had lower scores than patients without depression for self-efficacy, resilience and social support (*P* < 0.25).

**Table 2:** Psychological variables and depression and anxiety.

Variable	Without depression	depression	*t*	*P*	Without anxiety	anxiety	*t*	*P*
	Mean ± SD	Mean ± SD			Mean± SD	Mean± SD		
Stress	16.61±4.99	20.48±5.87	-4.536	0.000	16.88±5.08	22.03±5.87	4.796	0.000
Self-efficacy	26.91±5.81	24.02±7.26	2.852	0.005	26.37±5.82	25.00±8.88	1.050	0.295
Resilience	59.79±14.96	54.92±18.58	1.873	0.063	59.25±15.58	54.26±18.44	1.509	0.133
Social support	60.55±11.06	56.04±12.90	2.391	0.018	59.98±11.35	55.85±13.30	1.715	0.088

According to the Table 2, there was a statistical difference in the average scores for stress, resilience and social support between patients with anxiety and patients without anxiety. Patients with anxiety had significantly higher scores than patients without anxiety for stress (*P* < 0.001) and had lower scores than patients without depression for resilience and social support (*P* < 0.25). there were no significant differences in the average scores for self-efficacy between patients with anxiety and patients without anxiety.

### Logistic regression analysis for depression

The influencing factors for depression were shown in Table 3. There was a significant positive correlation between stress and depression, indicating that patients with high stress were more likely to develop depression (OR = 1.149, 95% CI: 1.055–1.253). There was a significant negative correlation between social support and depression, patients with a high social support were less likely to develop depression than those with a low social support (OR = 0.964, 95% CI: 0.931–0.997).

**Table 3:** Logistic regression analysis of the factors affecting the prevalence of depression.

Predictors	Reference	βeta	OR	95% CI	*P*
Gender					
Woman	Man	0.455	1.576	0.737–3.372	0.241
Diabetes history					
Yes	No	0.703	2.020	0.952–4.285	0.067
Other chronic disease					
Yes	No	0.586	1.796	0.850–3.794	0.125
Blood glucose control					
General	Good	0.225	1.253	0.391–4.019	0.705
Bad		0.556	1.744	0.542–5.614	0.351
Stress		0.139	1.149	1.055–1.253	0.001
Self-efficacy		-0.034	0.967	0.902–1.037	0.343
Resilience		0.007	1.007	0.978–1.038	0.635
Social support		-0.037	0.964	0.931–0.997	0.035

### Logistic regression analysis for anxiety

The influencing factors for anxiety were shown in Table 4. There was a significant positive correlation between family history of diabetes and anxiety. Patients with family history of diabetes were 5.79 times more likely to develop anxiety than patients without family history of diabetes (OR = 5.789; 95% CI: 1.856–18.057). Patients with other chronic disease were 4.31 times more likely to develop anxiety than patients without other diseases (OR = 3.309; 95% CI: 1.485–12.508). Stress was positively correlated with anxiety, and patients with high stress were at a higher risk of developing anxiety (OR = 1.278; 95% CI: 1.120–1.457). There was a significant negative correlation between social support and anxiety, indicating that patients with high social support were less likely to develop anxiety (OR = 0.947; 95% CI: 0.900–0.995).

**Table 4:** Logistic regression analysis of the factors affecting the prevalence of depression.

Predictors	Reference	*β*	OR	95.0% CI	*P*
Gender					
Man	Woman	0.506	1.658	0.563–5.161	0.359
Age					
45–60	< 45	0.077	1.080	0.294–3.966	0.908
> 60		-1.219	0.296	0.069–1.269	0.101
Residence					
Rural	Urban	0.432	1.541	0.532–4.464	0.426
monthly income					
≤ 4000	> 4000	1.143	3.137	0.861–11.428	0.083
Diabetes history					
Yes	No	1.756	5.789	1.856–18.057	0.002
Other chronic disease					
Yes	No	1.461	4.309	1.485–12.508	0.007
Stress		0.245	1.278	1.120–1.457	0.000
Social support		-0.055	0.947	0.900–0.995	0.032
Resilience		0.007	1.007	0.971–1.045	0.703

## Discussion

In this study, it was found that the overall mental health among DR patients was not optimistic. Among all the patients, there were 50 patients with depression symptoms, accounting for 25%, 27 patients with anxiety symptoms, accounting for 13.5%, and 23 patients with anxiety and depression simultaneously, accounting for 11.5%. These prevalence rates were higher than those reported in the general population [39]. In our study, the prevalence of depression was higher than Wang^’^s study [40]. However, another study found that the prevalence of depression and anxiety in patients with diabetes was higher than that in our study [41]. The difference in prevalence may be due to the difference in measurement tools and population, although there is no significant difference between the prevalence of depression and anxiety among diabetic people, there is a significant difference between the prevalence of depression and anxiety and the general population. Therefore, the psychological status among DR patients still needs to be paid attention to. In addition, it can be seen that anxiety and depression symptoms are likely to co-morbid, which is consistent with Poulsen’s study [42]. This phenomenon should be noticed because co-morbid anxiety and depressive disorders tended to have severe symptoms, poorer outcomes and greater use of healthcare resources than those with a single disorder [43].

In our study, compared with patients without family history of diabetes, the level of anxiety tended to be higher in patients with family history of diabetes. A study found adults with risk for Type 2 diabetes (T2DM) report greater subjective stress than adults who do not have risk for diabetes [44]. In addition, studies have shown that the occurrence of DR is related to the family history of diabetes, but whether the family history is a factor that causes anxiety symptoms among DR patients remains to be verified [45]. Interestingly, in Rees G’s research, the severity of DR is positively correlated with depressive symptoms. However, this factor is not included in our research, which we should explore and improve [46].

Our study found that patients with other chronic diseases had the higher risk of anxiety than those without other chronic diseases. The results of the study were consistent with Bickett [47] et al. study, and anxiety is associated with other chronic diseases among those with type 2 diabetes mellitus [48]. There might be two main reasons for the phenomenon. First, Patients with other chronic diseases costed more direct and indirect medical care than patients without other chronic diseases, and caused more burden on economy and psychology [49]. Second, patients with other chronic diseases tended to have severe physiological pain and function disruption, and cause more serious anxiety than those with a single-diseases. This phenomenon is widespread in patients with hypertension [50], cardiovascular disease [51] and brain tumor [52].

In this study, stress is positively correlated with depression and anxiety among DR patients. A similar study conducted by Didarloo et al. also found the significant effects of stress on depression and anxiety among patients with diabetes [29]. Study shown that stress could induce a series of psychological and physiological changes including activation of hypothalamic-pituitary-adrenal (HPA) axis and sympathetic nervous system [53]. Furthermore, the activation of HPA axis is one of the commonest neurobiological changes in depressive and anxiety patients [54].

Our study reveals a negative relationship between social support and depression, which is consistent with the previous findings [19]. It is generally believed that social support is an important aspect of psychological adjustment in previous study [55]. Moreover, previous study’s have shown that social support had an indirect correlation with the psychological health of diabetic patients [56]. Also, in Kaholokula study, DR patients with a lower social support and a worse physical health are likely to experience a high probability of depression [57].

In contrast to the findings reported by Wojujutari [58] and Devarajooh [59], resilience and self-efficacy were not significantly correlated with depression and anxiety in our study. There might be two reasons for the result. First, we have not enough samples size (N = 200), and an insufficient sample size may lead to a negative result. Second, the effect of resilience and self-efficacy weakened in logistic regression after other variables added. Therefore, Study with a large sample size and accurate variables should be explore in the future research.

### Implication

Several importantly theoretical and practical implications emerged from the findings of this study. In theory, this study revealed that stress had a negative impact on mental health in patients with DR. And social support had a protective effect on depression and anxiety among DR patients. In practice, the high prevalence of negative emotion in China DR patients (25.2%) should receive sufficient attention in Chinese medical setting; second, it was important for physicians and nurses to pay more attention to alleviating depression and anxiety among DR patients through providing more support; third, it was necessary to strengthen the education among DR patients on their knowledge of the disease for reducing their stress; last but most importantly, it was necessary to improve basic public health services and provide a good medical environment for patients.

### Limitations

First, the use of self-filled questionnaires to understand the basic conditions of patients and to evaluate patient’s mental health is defective, which may lead to data recall bias, and in some cases, the questionnaires do not truly reflect the extent of patient’s psychological conditions. Second, the study was a cross-sectional design and could not determine causality among variables. Third, as a confounding factor in this study, the relationship between family history of diabetes and anxiety symptoms among DR patients needs further study. Finally, our study was conducted in He Eye Specialist Hospital, China. The result is limited to Chinese DR patients, and the representativeness is difficult to spread.

## Conclusions

In summary, part of Chinese DR patients suffered from depression and anxiety symptoms in our study. Our study found that social support was negatively correlated with depression and anxiety in Chinese patients with DR. In addition, our result indicated that stress and other chronic diseases were risk factor for depression and anxiety among DR patients. Therefore, the strategies to prevent and treat depression and anxiety in DR patients should focus on increasing the social support and reducing the stress in patients.

## Supporting information

S1 Data (XLS)
